# Profiling adolescent participation in wildlife activities and its implications on mental health: evidence from the Young-HUNT study in Norway

**DOI:** 10.3389/fpubh.2025.1517089

**Published:** 2025-03-12

**Authors:** Skender Elez Redzovic, Ingvill Jeanette Hektoen Johansen, Tore Bonsaksen

**Affiliations:** ^1^Department of Neuromedicine and Movement Science, Norwegian University of Science and Technology, Trondheim, Norway; ^2^Department of Health and Nursing Science, Inland Norway University of Applied Sciences, Elverum, Norway; ^3^Department of Health, VID Specialized University, Stavanger, Norway

**Keywords:** wildlife activities, adolescents, participation profile, mental distress, cross-sectional study, Trøndelag Health Study (HUNT), Norway

## Abstract

**Background:**

Adolescent mental health challenges are on the rise globally, and Norway is no exception. Wildlife activities (WAs) have been increasingly promoted as a potential measure to improve adolescent well-being. However, there is limited research on the extent of adolescent participation in these activities and its association with mental distress among Norwegian adolescents.

**Aim:**

This study has a twofold aim: to explore the degree of adolescent participation in WAs and to examine the association between this participation and mental distress.

**Methods:**

The study design was cross-sectional, using Young-HUNT data from Norway collected between 2017 and 2019. A total of 6,361 participants were included in the final sample. Participants were categorized based on their level of participation in WAs, and ordinal regression analysis was conducted to assess factors associated with their level of wildlife activity. Furthermore, multivariate linear regression analysis assessed the association between wildlife activity and mental distress.

**Results:**

The majority of participants reported low levels (34.6%) or medium levels (53.2%) of engagement in WAs. Adjusted analyses showed that higher levels of participation in WAs were associated with a range of factors, particularly female sex, having both parents born in Norway, and maintaining a medium or high level of physical activity. A weak yet statistically significant relationship was identified between higher levels of wildlife activity and increased mental distress among the participants.

**Conclusion:**

Various sociodemographic, lifestyle, and social factors influence the involvement in WAs. The observed weak but significant association between higher participation in WAs, and increased mental distress raises questions about the assumption that nature and WAs are universally beneficial for adolescent mental health. These results highlight the need for further research to explore the underlying mechanisms of this relationship. These findings also caution policymakers against making generalized claims about the mental health benefits of WAs without a deeper consideration of individual and contextual factors.

## 1 Introduction

Globally, increasing evidence indicates a rise in mental health challenges among children and adolescents aged 4 to 18 years over the past five decades (1, 2). Similarly, the findings from Norway's national cross-sectional Ungdata study show a consistent increase in the percentage of adolescents (13–19 years) experiencing mental health issues, such as depression and anxiety, in recent years (3, 4).

In accordance with current public health policies, participation in wildlife activities (WAs) is promoted as an effective and affordable health-promoting measure for adolescents from all socioeconomic backgrounds, as it positively influences their physical and mental health and wellbeing (5, 6). The Norwegian authorities define WAs as “staying and engaging in physical activity outdoors during leisure time with the aim of environmental change and experiencing nature.” (7). WAs encompass a broad spectrum of nature-related activities, which can be both structured (e.g., organized hiking) and unstructured (e.g., casual nature walks or free play). Additionally, the social context of WA—whether solitary or social (8)—may influence its mental health effects. For example, solitary WAs might provide different psychological benefits or challenges compared to social participation, where peer interactions play a significant role.

Participation in WAs among Norwegian adolescents is reported to be high and stable. Due to significant differences in sample sizes across surveys conducted over the past 20 years, a 2020 survey (9, 10) showed that 68.8% of children and adolescents engage in WAs weekly or several times a week. This high percentage suggests that many children participate in multiple activities and are generally active, even if they do not attend each activity weekly. Furthermore, 23.6% engage in WAs a few times a month, while 7.6% do so less than once a month or never [(9), p. 73]. However, participation in certain activities, such as skiing, has decreased, whereas involvement in others, such as hiking, has increased (11). Moreover, children and adolescents' free play in nearby nature settings does not seem to serve as an alternative to a goal-oriented and time-structured daily routine (12). Evidence indicates that participation in organized activities in Norway has increased, and WAs has also become more institutionalized (13). A wide range of individual and environmental factors influence participation in WAs. Among children and adolescents with low activity levels, factors such as low economic status, limited parental education, and immigrant background are particularly prominent (9).

While participation in WAs is widely recognized as an affordable preventive measure that does not impose pressure on the performance or results, there is a notable lack of empirical studies with high statistical power in this area. Additionally, many existing studies have merged samples of both children and adolescents. Given the shifts in activity patterns that commonly occur during adolescence, this complicates obtaining a clear understanding of adolescents' participation in WA. More knowledge about adolescents' participation is also necessary because WAs are unique as they attract participants who may not engage in organized sports (11, 13). Recent reports indicate that fewer children and adolescents have been participating in organized activities in recent years (4). Moreover, technological advances have drastically transformed activity patterns, especially among adolescents who are immersed in this new world. Research has shown that the use of digital technology predicts inactivity (14) and negatively impacts adolescents' mental health (15, 16). Thus, research into WAs appears particularly relevant in the current context of a digital culture.

The nature of the relationship between WAs and mental health remains unclear. According to the “Biophilia” hypothesis, everyone has an inherent connection with nature, which is beneficial and rooted in a strong genetic and evolutionary foundation. All humans have a fundamental need for contact with nature (17). The “Attention Restoration” theory (18, 19) and the “Stress Recovery” theory (20, 21) are based on the idea that exposure to nature has a positive effect on mental health through attention restoration, stress reduction, and enhancement of wellbeing.

Empirical studies have shown that exposure to natural environments has a medium-to-large effect on both increasing positive and decreasing negative affect (22). A recent study found that individual factors such as childhood and current nature exposure, nature connectedness, gender, or age did not predict immediate affective responses to nature (23). These findings suggest that humans may have an innate, hard-wired tendency to evoke positive emotions in response to nature, while individual factors significantly influence affective responses to urban scenes. Another study revealed that images of natural settings elicited more intense positive emotional responses and greater feelings of relaxation compared to representations of constructed and urban environments. Although nature connectedness and preference played a role in moderating these effects, they did not completely account for the emotional advantages associated with exposure to nature (24).

Moreover, studies indicate that participation in WAs reduce stress, increase self-esteem, improve mood, enhance self-efficacy and resilience, decrease symptoms of anxiety and depression, and boost academic and cognitive performance, which are all linked to better school functioning (25–28).

Although some qualitative studies and research confirm the effects of a Nordic form of wilderness therapy (29–31), the relationship between adolescents' participation in WAs and mental health in the Norwegian context has yet to be explored. Since WAs are grounded in specific ecological, historical, and cultural contexts, international research may not be entirely applicable.

The primary objective of this study is to explore adolescents' involvement in WAs and its connection to their mental health by utilizing population data from the Young-HUNT study conducted in Norway. Through this approach, we aim to provide knowledge to support authorities in shaping public health practices. To achieve this knowledge, two specific aims have been set. First, we aim to develop a profile characterizing adolescents aged 13–19 years who exhibit high, moderate, and low levels of participation in WA. Second, the study investigates whether participation in WAs WA is associated with psychological distress. We hypothesize differences in gender, ethnicity, socioeconomic background, lifestyle, media use, and school performance between those with varying levels of participation. Additionally, we expect that higher levels of participation in WAs will be associated with lower levels of psychological distress.

## 2 Methods

### 2.1 The Norwegian context

According to national statistics, the majority of adolescents reside in nuclear families (32). The vast majority feel that they have good friends, have close and trusting relationships with their parents, and report high satisfaction with Norwegian schools. Few experience significant mental health issues, and only a minority report feeling a high degree of loneliness (4). The educational system in Norway consists of lower secondary school (ages 13 to 16) and upper secondary school (ages 16 to 19), with approximately 95% of adolescents attending public schools. Additionally, students in lower secondary school have the right to attend the school closest to their homes. In upper secondary school, adolescents can opt for either a general studies program or vocational education, allowing many adolescents in the same geographical vicinity to attend the same school, regardless of their socioeconomic status. Furthermore, national reports suggest that approximately 50% of Norwegian adolescents engage in organized leisure activities (4), although the types of activities differ between those living in urban and rural areas of Norway (3).

#### 2.1.1 Study population and sample

The sample comprised adolescents aged 13 to 19 years who participated in the Young-HUNT4 survey conducted in Norway (33) (*n* = 8,066, response rate = 76%). The Young-HUNT4 survey, conducted from 2017 to 2019, represents the fourth iteration of the Young-HUNT studies. The study employs a cross-sectional design, utilizing data from the questionnaire in the survey, which included the youth population of the former county Nord-Trøndelag in central Norway. The population of this county is ethnically and socioeconomically homogeneous, primarily inhabiting rural areas and small urban centers. Nevertheless, the county is considered to be representative of Norway in terms of both demographics and geography. The Young-HUNT study includes students from 66 schools (33). Invitations were sent to participants through detailed letters providing comprehensive information about the study and data usage. Informed consent was obtained directly from adolescents aged 16 years and older, while parents provided consent for those below this age threshold. Specially trained nurses conducted interviews and measurements during visits to the schools. Students absent on the day of the questionnaire were encouraged to complete it during the nurses' visits. According to records from the county school authorities, adolescents not attending school were invited to participate in the study via mail.

Participants reporting movement restrictions (*n* = 79), breathing problems (*n* = 281), juvenile arthritis (*n* = 178), and pain when walking more than one kilometer (*n* = 905) were removed from the sample because of their physical conditions that would impact their ability to perform outdoor activities. There was an overlap between the categories of health problems. Inclusion in the sample also required that participants respond to the main outcome variable in the study, namely psychological distress. After the removal of those ineligible according to the above criteria, the study sample consisted of 6,361 adolescents (78.9% of the original sample). The number of participants varied across analyses due to some missing responses on employed variables, and n is reported for each analysis.

### 2.2 Measurements

#### 2.2.1 Sociodemographic information

Age was recorded in years, and gender was categorized as boys or girls. Relative socioeconomic status (SES) was assessed with the question: “How well off do you believe your family is compared to most others?” Response options included “worse off” (1), “like most others” (2), and “better off” (3). Country of birth was categorized as “born in Norway” or “born in a country other than Norway.” Parents' country of birth was first assessed in the same way, and then the information was transformed into a variable with three categories: none of the parents born in Norway (0), one parent born in Norway (1), and both parents born in Norway (2). Parents were also reported as divorced (1) or not divorced (0).

#### 2.2.2 Wildlife activity

Participation in WAs and a variety of other activities was assessed, beginning with the question: “How often do you engage in these physical activities?” Wildlife was included as one of the activities, with hiking and cross-country skiing as examples. The response options were originally never/rarely, 2–3 times per month, once a week, 1–3 times per week, and 4 times per week or more. These categories were then reorganized into never or rarely (low level; 1), between 2–3 times per month and once a week (medium level; 2), and 1–3 times per week or more often (high level; 3).

#### 2.2.3 Lifestyle

Self-reported weight and height were used to calculate body mass index (BMI) values. Alcohol consumption was evaluated with the question, “Do you occasionally drink alcohol? (Alcoholic beer, wine, soda/cider, liquor, or home-brewed)” and response options of yes (1) or no (0). Illicit drug use was assessed using two questions: “Have you ever tried cannabis, marijuana, or other cannabis-like substances?” and “Have you ever tried other drugs, such as amphetamines, cocaine, or ecstasy?” Both questions provided response options of yes or no. Based on the responses to these questions, a new variable was created to distinguish between individuals who had tried any illegal drug (1) and those who had not (0).

Physical activity was assessed using the question: “Outside school hours: How often do you engage in sports or physical activity to the point of being short of breath and/or breaking a sweat?” Response options included never, less than once a week, once a week, 2–3 times per week, 4–6 times per week, and every day. Subsequently, answers to this question were reclassified into three categories: low level (once a week or less; 1), medium level (2–3 times per week; 2), and high level (4–6 times per week or more; 3).

#### 2.2.4 Support from adults

Support from adults was assessed using one question: “Do you feel safe with and can you seek support from at least one adult?” Response options were categorized as “no” (0), “yes, sometimes” (1), and “yes, always” (2).

#### 2.2.5 Media use

Gaming on weekdays was evaluated with one question: “In your spare time, how many hours per day do you typically spend on gaming (on computer, game console, tablet, phone, etc.)?” The use of social media and online browsing/chatting on weekdays was measured with the question: “In your spare time, how many hours per day do you usually spend on social media and/or browsing/chatting online?” The consumption of TV and other screen-based entertainment was assessed with the question: “In your spare time, how many hours per day do you typically spend watching TV or engaging in other screen-based entertainment?” All questions featured response options: not at all (1), <½ h per day (2), between ½ and 1 h per day (3), 2–3 h per day (4), 4–6 h per day (5), or approximately 7 h or more per day (6).

#### 2.2.6 School-related factors

Motivation for performing well in school was evaluated with the question, “It is important for me to excel academically.” Response options included “completely disagree” (1), “disagree” (2), “agree” (3), and “completely agree” (4).

School absence was assessed by asking, “How long have you been absent from school during the past 12 months?” Response options were <1 week (1), 1–3 weeks (2), and more than 3 weeks (3).

Assessing the management of school demands involved a question about the student's experiences during the past week: “How well do you tackle the school demands?” The response options were “very poorly (1),” “quite poorly (2),” “neither well nor poorly (3),” “quite well (4),” and “very well (5).”

#### 2.2.7 Mental distress

We incorporated the Symptoms Checklist 5 (SCL-5), a tool that assesses both depressive and anxiety symptoms through five statements. Adolescents were asked about their experiences over the past 2 weeks, specifically whether they “Felt scared or anxious,” “Felt tense or hurried (restless),” “Felt hopeless when thinking about the future,” “Felt down and sad,” or “Worried too much about various things.” To measure mental distress, we used a scale adapted from the Hopkins Symptom Checklist, which has been previously utilized for such evaluations and shown compatibility with comprehensive, non-abbreviated instruments (34). The SCL-5 items provide four response options: “Not bothered,” “Slightly bothered,” “Fairly bothered,” and “Very bothered,” rated from 1 to 4, where higher scores indicate a greater symptomatic burden. In this study, we calculated the mean of the five items as the scale score, employing a recommended cutoff score of 2.0 to identify depressive/anxiety symptoms (34).

### 2.3 Statistical analysis

Participation in WAs was categorized into three levels (low, medium, and high), and differences in characteristics between adolescents in the three respective groups were assessed using chi-Squared tests for categorical variables and one-way analyses of variance (ANOVAs) for continuous variables. To adjust for potential confounding factors, ordinal regression analysis was employed to evaluate the direct relationships between independent variables and levels of wildlife activity. Independent variables that showed a statistically significant association with the outcome (level of wildlife activity) in unadjusted analyses were included in the multivariate regression analysis. Estimates of association were reported along with their 95% confidence intervals (CIs) and *p*-values.

Furthermore, we performed linear regression analysis to assess the associations between independent variables and levels of mental distress (SCL-5 score). Again, independent variables showing a statistically significant association with the outcome in unadjusted analyses were carried over from single regression analysis to multivariate analysis. Standardized beta (β) values were utilized to measure the strength of associations, and statistical significance was established at a *p*-value of < 0.05.

In the multivariate model, multicollinearity was assessed using the variance inflation factor (VIF). None of the VIFs exceeded 2.23, indicating no multicollinearity. The standardized residuals ranged from −3.36 to 4.55, slightly exceeding the preferred range (−3, 3) (35). However, the distribution of the residuals was visually inspected and deemed to have no significant deviation from the normal distribution. The scatterplot of standardized predicted values plotted against standardized residuals displayed no distinct pattern, indicating that the regression model was well-fitted across the entire spectrum of SCL-5 scores (homoscedasticity).

## 3 Results

### 3.1 Characteristics of adolescents with different profiles of wildlife activity

Of those who answered the question regarding wildlife activity, 34.6% were classified as having a low level of participation. More than half of the participants (53.2%) had a medium level of participation, while 9.6% had a high level of participation in WAs.

Table 1 shows the characteristics of participants categorized within each of the three profiles of wildlife activity: low, medium, and high. Regarding sociodemographic factors, adolescents exhibiting a high level of wildlife activity had a lower mean age; they were predominantly girls and more frequently perceived their family economic status as similar to that of others. In contrast, adolescents displaying a low level of wildlife activity were more likely not to have been born in Norway, had one or both parents born in a different country, and were more often from divorced parents.

**Table 1:** Characteristics of adolescents within three profiles of wildlife activity.

**Characteristics**		**Profile of wildlife activity**			
	***n*** **included**	**Low**	**Medium**	**High**	***p***
Total sample	6,190	2,199 (34.6)	3,381 (53.2)	610 (9.6)	
**Sociodemographic variables**					
Mean age (SD)	6,190	16.6 (1.7)	16.4 (1.8)	16.3 (1.8)	<0.001
Gender n (%)	6,190				
Girls		895 (29.1)	1,822 (59.3)	355 (11.6)	<0.001
Boys		1,304 (41.8)	1,559 (50.0)	255 (8.2)	
Perceived family SES	6,140				
Worse off *n* (%)		169 (38.9)	229 (52.6)	37 (8.5)	0.02
Like others *n* (%)		1,540 (34.7)	2,422 (54.6)	473 (10.7)	
Better off *n* (%)		461 (36.3)	710 (55.9)	99 (7.8)	
Country of birth	6,189				
Not Norway		218 (57.4)	139 (36.6)	23 (6.1)	<0.001
Norway		1,981 (34.1)	3,242 (55.8)	586 (10.1)	
Parents' country of birth	6,134				
None from Norway		211 (61.3)	115 (33.4)	18 (5.2)	<0.001
One from Norway		159 (40.3)	191 (48.4)	45 (11.4)	
Both from Norway		1,804 (33.4)	3,048 (56.5)	543 (10.1)	
Divorced parents	6,140				
Not divorced *n* (%)		1,401 (33.5)	2,361 (56.5)	414 (9.9)	<0.001
Divorced *n* (%)		769 (39.2)	1,004 (51.1)	191 (9.7)	
**Lifestyle**					
Mean BMI (SD)	5,769	22.3 (4.3)	22.1 (3.9)	22.3 (4.3)	0.07
Alcohol use	6,173				
Not occasional drinker *n* (%)		1,109 (34.2)	1,792 (55.3)	337 (10.4)	0.06
Occasional drinker *n* (%)		1,081 (36.8)	1,584 (54.0)	270 (9.2)	
Illicit drug use	6,126				
Have not tried illicit drugs		1,979 (34.6)	3,162 (55.2)	584 (10.2)	<0.001
Have tried illicit drugs		195 (48.6)	191 (47.6)	15 (3.7)	
Physical activity	6,175				
Low level		710 (43.2)	788 (48.0)	145 (8.8)	<0.001
Medium level		737 (33.6)	1,229 (56.0)	227 (10.4)	
High level		744 (31.8)	1,358 (58.1)	237 (10.1)	
*Support from adults*	6,040				
No		211 (45.4)	205 (44.1)	49 (10.5)	<0.001
Yes, sometimes		351 (36.7)	508 (53.1)	98 (10.2)	
Yes, always		1,581 (34.2)	2,596 (56.2)	441 (9.5)	
**Media use**					
Mean hours gaming (SD)	6,114	3.3 (1.5)	2.9 (1.4)	2.9 (1.4)	<0.001
Mean hours SoMe/chatting (SD)	6,092	4.1 (1.2)	3.9 (1.1)	3.8 (1.1)	<0.001
Mean hours TV/screen (SD)	6,027	3.3 (1.2)	3.2 (1.0)	3.1 (1.1)	<0.001
**School-related factors**					
Mean performance motivation (SD)	5,969	3.39 (0.70)	3.52 (0.62)	3.47 (0.67)	<0.001
Mean tackling school demands (SD)	5,989	3.81 (0.89)	3.89 (0.84)	3.90 (0.91)	<0.001
School absence past year	5,986				
<1 week		1,533 (34.4)	2,483 (55.7)	443 (9.9)	0.15
1–3 weeks		486 (36.6)	721 (54.3)	120 (9.0)	
More than 3 weeks		83 (41.5)	97 (48.5)	20 (10.0)	

*p*-values are derived from ANOVAs for continuous variables and Chi-squared tests for categorical variables. SES, socioeconomic status; SD, standard deviation; SoMe, social media; BMI, body mass index.

Regarding lifestyle factors, adolescents with a high level of wildlife activity more frequently reported higher levels of physical activity and were less likely to have tried using illicit drugs. Group differences in BMI and alcohol use did not achieve statistical significance.

With regards to social support, adolescents reporting no support from at least one adult were more likely to have a low level of wildlife activity. However, adolescents in the low support group were also overrepresented among those with a high level of wildlife activity, whereas those reporting to have adult support more often had a medium level.

Adolescents spend approximately 1 h more daily on social media and online chatting than on other forms of media. For all forms of media use, those with a low level of wildlife activity spent more hours than their counterparts.

Among school-related factors, those with a medium level of wildlife activity were most motivated to perform at school, while those with the lowest levels of wildlife activity also reported the lowest ability to tackle school demands. Differences in school absence during the past year were not significant between the groups.

#### 3.1.1 Adjusted associations with wildlife activity

Table 2 shows the results from the multivariate ordinal regression analysis, which demonstrated adjusted associations between each independent variable and wildlife activity. Higher age was associated with a lower level of wildlife activity, while girls had a higher level than boys. Adolescents whose parents were born outside of Norway had a lower level of wildlife activity than those whose parents were both born in Norway.

**Table 2:** Ordinal regression analysis showing adjusted associations between independent variables and wildlife activity participation.

**Independent variables**	**Wildlife activity**		
	**Estimate**	**95% CI**	***p***
Age	−0.04	−0.07 to −0.01	0.01
**Gender**			
Girls	0.49	0.37–0.61	<0.001
Boys	Reference		
**Perceived family SES**			
Worse off	0.02	−0.22–0.25	0.89
Like others	0.07	−0.06–0.20	0.30
Better off	Reference		
**Country of birth**			
Other than Norway	−0.16	−0.55–0.22	0.41
Norway	Reference		
**Parents' country of birth**			
None born in Norway	−0.87	−1.27 to −0.47	<0.001
One born in Norway	−0.14	−0.36–0.08	0.08
Both born in Norway	Reference		
**Divorced parents**			
Not divorced	0.09	−0.03–0.21	0.12
Divorced	Reference		
**Illicit drug use**			
Have not tried illicit drugs	0.33	0.10–0.55	<0.01
Have tried illicit drugs	Reference		
**Physical activity**			
Low level	−0.23	−0.37 to −0.09	0.001
Medium level	−0.02	−0.14–0.11	0.79
High level	Reference		
**Support from adults**			
No	−0.25	−0.45 to −0.04	0.02
Yes, sometimes	0.03	−0.12–0.17	0.73
Yes, always	Reference		
Gaming	−0.10	−0.14 to −0.06	<0.001
SoMe/online chatting	−0.14	−0.19 to −0.09	<0.001
TV/screen entertainment	−0.03	−0.08–0.02	0.27
School performance motivation	0.14	0.06–0.22	<0.001
Ability to tackle school demands	0.03	−0.03–0.10	0.35

For continuous variables, higher values indicate higher levels. SES, socioeconomic status; SoMe, social media.

Adolescents who had never tried using illicit drugs exhibited higher levels of wildlife activity compared to those who had. Additionally, those with low physical activity also demonstrated lower levels of wildlife activity than their peers with high physical activity levels. Those who reported lacking adult support had lower levels of wildlife activity than those who received support from an adult. Increased gaming and engagement with social media and online chatting were linked to lower wildlife activity levels. Adolescents motivated to perform well in school showed higher levels of wildlife activity compared to those with lesser motivation. The other variables included in the model did not exhibit significant associations with the outcome. The most notable effect sizes were observed for female sex (0.49) and for adolescents with both parents born abroad (−0.87).

#### 3.1.2 Psychological distress and its associated factors

**Figure 1:**
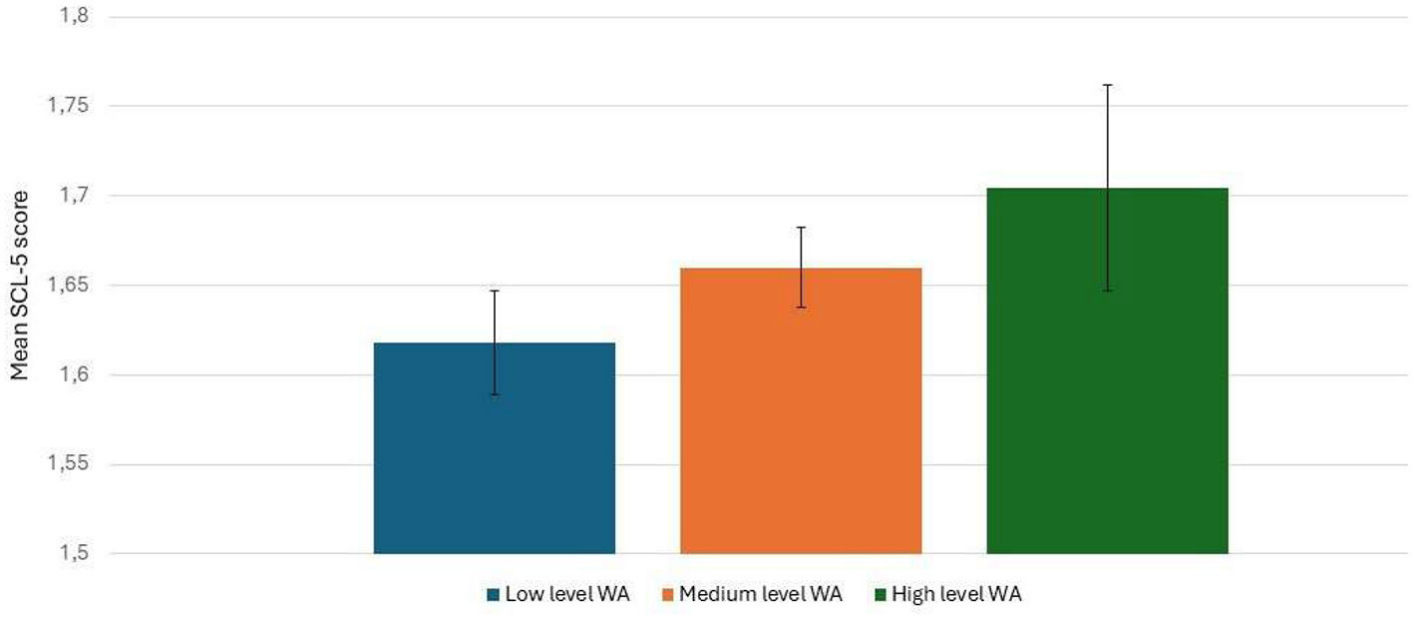
Differences in mental distress (mean SCL-5 scores) by levels of wildlife activity. Error bars represent 95% confidence intervals.

The results of the single and multiple linear regression analyses, demonstrating unadjusted and adjusted associations with mental distress (SCL-5 scores), are displayed in Table 3. The unadjusted differences in mental distress by levels of wildlife activity are shown in Figure 1, showing that more active adolescents experienced higher levels of distress. In the multivariate model adjusted for all included variables, a weak but statistically significant positive association remained between higher levels of wildlife activity and mental distress. Two associations displayed moderate effect sizes: girls reported higher levels of mental distress than boys (β = −0.27), and those who felt more capable of handling school demands had lower levels of mental distress than their peers (β = −0.32). Although most of the other included associations achieved statistical significance, they were weak (i.e., β ≤ 0.11) to negligible.

**Table 3:** Single and multivariate linear regression analyses showing both unadjusted and adjusted associations between the independent variables and SCL-5 scores (*n* = 5,567).

**Independent variables**	**Single analysis**		**Multivariate analysis**	
	**Std**. β	***p***	**Std**. β	***p***
Age	0.16	<0.001	0.08	<0.001
Gender	−0.36	<0.001	−0.27	<0.001
Perceived family SES	−0.12	<0.001	−0.03	<0.01
Divorced parents	0.09	<0.001	0.03	<0.01
Born in Norway	−0.00	0.95	–	–
Parents born in Norway	−0.03	0.02	−0.02	0.09
Gaming	−0.07	<0.001	−0.02	0.16
SoMe and online chatting	0.24	<0.001	0.10	<0.001
TV/screen entertainment	0.10	<0.001	0.03	0.03
School performance motivation	0.01	0.35	–	–
Ability to tackle school demands	−0.42	<0.001	−0.32	<0.001
Support from adults	−0.17	<0.001	−0.11	<0.001
Illicit drug use	0.12	<0.001	0.06	<0.001
Physical activity	−0.16	<0.001	−0.07	<0.001
Wildlife activity	0.04	<0.01	0.05	<0.001
**Explained variance**			**32.0%**	**<0.001**

SES, socioeconomic status; SoMe, social media. The reference values for categorical variables are female sex, lower SES, no divorced parents, not born in Norway, and no illicit drug use. For continuous variables, higher values indicate an older age; more parents born in Norway; increased time spent gaming, using SoMe and online chatting, and engaging in TV/screen entertainment; higher motivation for school performance; greater perceived ability to meet school demands; more certainty about support from adults; as well as higher levels of physical activity and wildlife activity.

## 4 Discussion

To the best of our knowledge, this is the only study in the Norwegian context aiming to understand adolescents' participation in WAs and its association with mental distress.

This study showed how various factors can be used to develop profiles of adolescents' participation in WAs. Several factors related to sociodemographic background, lifestyle, social support, media use, and school performance were identified as covariates of wildlife involvement. Furthermore, the study revealed—contrary to expectations—a weak but statistically significant relationship between higher levels of wildlife activity and elevated levels of psychological distress. Additionally, a range of other factors were significantly associated with psychological distress among adolescents, most notably female sex and a lower ability to meet school demands.

Our results indicate that adolescent participation in WAs is lower compared to Statistics Norway's estimates. Among our respondents, 34.6% participated at low levels (none to two or three times a month), which aligns with existing reports. However, while current estimates suggest that 40%−58% engage in WAs several times a week (9, 10), our results revealed only 9.6%. Although other studies report that 10%−25% participate once a week, our findings showed that approximately 53% do so. Our findings are more consistent with an earlier report indicating a decline in outdoor recreation among young people aged 16–24 years from 1970 to 2011 (36, 37).

This discrepancy could be due to the division of existing statistics into two broad age groups: children aged 6 to 15 years and adults aged 16 and older (9, 10). Recall bias and a skewed sample in previous studies may also contribute to this difference, as earlier investigations often relied on parents answering on behalf of their child or adolescent. Moreover, in line with some previous studies (14, 38), our findings suggest that those who participate less in WAs tend to spend more time on media. This trend may be linked to evolving activity patterns and cultural shifts resulting from technological advancements in society. Nevertheless, our study highlights the need for further research to better understand the frequency of adolescent participation in WA.

Furthermore, our findings indicate a consistently lower rate of participation in WAs among boys, which contrasts with the results from previous studies suggesting only minor differences (9). It appears that boys may engage more in structured sports (39) and digital media, such as gaming (40), which could contribute to diminished involvement in physical activities. In alignment with prior research (9, 11), our findings reveal that participants with parents who were not born in Norway show significantly lesser participation in physical activities, implying that cultural factors may play a role in this engagement (41, 42). This may be associated with the perceived value and attractiveness of such activities or the extent of expectations, support, and encouragement received from parents (13, 43). Our results suggest that adolescents lacking parental support are likely to participate less in physical activities. This is similarly observed in adolescents from divorced families, who may experience diminished support in engaging in activities that necessitate additional parental time and effort, such as transportation. Furthermore, this trend may be intensified by the generally lower economic standing of these households. Consistent with previous findings, our results indicate that adolescents from families perceived to have an economic status comparable to that of their peers tend to exhibit greater activity levels in physical pursuits (10, 36).

Our findings paint a concerning picture of adolescents with low engagement in WA, highlighting a vulnerable background. These adolescents often come from families with poorer economic conditions, parents who were born outside of Norway, and divorced households. They also demonstrate lower physical activity levels and are more likely to experiment with illicit drugs. Additionally, they are more inclined to lack a person they can turn to for support, spend more time using media, and demonstrate lower motivation in school. While these results are not entirely unexpected, they are still alarming, as adolescents with such characteristics may benefit the most from participating in WA. Surprisingly, despite WAs being both affordable and non-demanding in terms of performance or outcomes, adolescents from vulnerable backgrounds remain underrepresented (5, 6).

The most unexpected finding in our study is the positive association between increased participation in WAs and heightened mental distress. This contrasts sharply with much of the previous research and theories that emphasize nature's mental health benefits (25, 26, 28, 29, 44, 45). However, in a systematic review, limited evidence suggested beneficial associations with greater mental wellbeing in children and lower levels of depressive symptoms in adolescents (46). The majority of existing research on nature and mental health focuses on urban populations in North America, Europe, and Australia (47), where access to green spaces is restricted, potentially making exposure to nature more impactful. To our knowledge, there are no population studies from comparable regions, such as the Nordic countries, Canada, or Greenland, to benchmark our findings. This lack of comparable research highlights a gap in the literature, particularly in areas where nature is a common part of daily life. Thus, our findings may be influenced by the unique environmental and cultural context of our study population, drawn from a rural region of Norway, where green spaces abound. The relationship between nature and wellbeing might be more complex in countries like the Nordic countries, Canada, and Greenland, where nature is woven into daily life. For example, research from Finland indicates that frequent and ingrained interactions with nature contribute to a cultural baseline of nature exposure (8). Similarly, WAs have deep cultural roots in Norway (41, 42), raising the possibility that increased engagement with nature may not deliver the same restorative benefits seen in urban populations.

Another possible explanation is selection bias—adolescents who experience higher mental distress may be more likely to turn to nature for relief. Given the strong public health message that “nature is good for you,” youth experiencing symptoms of stress, anxiety, or depression might engage in WAs as a coping mechanism. Additionally, since friendships and peer relationships are vital to young people's mental health, those with fewer social connections may seek solace in nature to alleviate feelings of loneliness. However, if WAs serve primarily as a form of emotion-focused coping (48)—helping adolescents manage emotions without addressing underlying stressors—it may not provide lasting relief. This aligns with Lazarus and Folkman's stress and coping theory (49), which distinguishes between emotion-focused coping (which manages distress) and problem-focused coping (which addresses the source of stress). Studies have shown that adolescents cope with life stressors in different ways, often varying by gender. Females typically rely on social support and emotional coping strategies, whereas males are more inclined to use problem-focused approaches (50, 51). If adolescents use nature as an escape without resolving their issues, their levels of distress may persist.

Another key factor is the social context of WA participation. While WAs are often framed as a health-promoting activity, it is essential to consider whether adolescents engage in WAs alone or with others. However, our study does not include measures that address this aspect. Research suggests that social relationships are critical for adolescent wellbeing. If WA participation occurs primarily in solitary settings, it might contribute to increased feelings of loneliness and rumination, particularly for those already struggling with mental health problems. On the other hand, structured, socially integrated WAs (e.g., organized hiking groups) may provide protective effects. The higher observed distress levels among high WA participants may reflect solitary engagement rather than the negative effects of WAs themselves.

This complexity challenges existing nature-related theories. The Biophilia hypothesis posits an inherent human connection to nature (17), yet our findings suggest that mere presence in nature does not universally enhance wellbeing. Instead, the effects of WAs may depend on how and why individuals engage in it. Attention Restoration and Stress Recovery Theory propose that nature facilitates cognitive restoration and reduces stress (18–21). However, these benefits may not manifest if WAs are used merely as an emotional escape without constructive problem-solving. Without effectively addressing the sources of mental distress, WAs might not contribute to the expected stress reduction and restorative benefits. Moreover, the type of activity (structured vs. unstructured), social context (solitary vs. social), and nature connectedness can moderate these effects. For example, the stress-reducing benefits of WAs might differ based on whether the activity is performed alone or with others, as social aspects can significantly influence emotional responses and mental health outcomes.

Furthermore, digital culture may interact with WA participation in complex ways. Adolescents who face challenges with social interactions might seek refuge in nature, but this does not necessarily alleviate feelings of isolation. Engaging in social media and online chatting to connect with others may prove unhelpful, as such connections can feel less genuine, less meaningful, and more superficial than desired (52). Additionally, increased time spent on social media may come at the expense of real-life interactions (53). Paradoxically, these online efforts to connect could lead to greater mental distress instead of providing relief. While taking a break from challenges might be necessary, adolescents need to develop a capacity for problem-focused coping; that is, the ability to address actual problems to change the sources of stress (54).

### 4.1 Strengths and limitations

A key strength of this study is the large number of respondents, which provides robust data. The sample represents the Norwegian context, making the findings highly relevant. Additionally, the exclusive focus on adolescents ensures that the insights are specific to this age group. Psychological distress was measured using the well-established Symptom Checklist (SCL), which adds credibility to the results.

However, there are limitations. The sample is drawn from rural areas and small towns, where adolescents are regularly exposed to nature. Physical activity was assessed through questions specifically designed for this study, which may complicate comparisons with established measurement scales used in other research. Additionally, the measurement of WAs in this study differs slightly from that used in other studies, which may limit comparability. In the Young-HUNT 4 survey, the question about WAs is included as a subcategory of various physical activities. For example, one question asks how often participants engage in endurance sports (e.g., cross-country skiing). Such activities could also be classified as WAs but are not categorized in the survey. Moreover, the extent to which participants engaged in WAs as part of organized activities (e.g., organized hiking) or through unstructured activities (e.g., casual nature walks) remains unknown. Similarly, there is no information on whether WAs were performed alone or in a social context, and these aspects of WAs could affect mental health outcomes. Finally, as a cross-sectional study, it cannot determine causality, leaving the direction of the relationship between WAs and psychological distress unclear.

### 4.2 Implications for practice and further research

While WAs are often advocated for its positive effects, our results indicate that its impact on mental health is more complex than commonly believed. Authorities should exercise caution when making broad assumptions about the benefits of WA, ensuring they account for individual differences in coping strategies, levels of social engagement, and the emotional context surrounding participation. Furthermore, authorities should encourage additional research to deepen the understanding of these factors and guide more targeted interventions.

Given the unexpected findings concerned with participation rates, more in-depth research is needed to understand the extent and nature of adolescent involvement in WA. The decline in WA participation warrants further investigation, with future studies focusing on identifying the underlying causes, particularly among boys and adolescents with parents born abroad.

The negative impact of social media on WA participation is also a growing concern, as it reduces physical activity and time spent in nature. Future research and interventions should focus on mitigating this impact and further investigating the interplay between technology use and WA.

Ultimately, our study indicates that the positive link between WAs and mental health should not be taken for granted. The effects of factors such as the quality of green spaces, the duration and frequency of nature exposure, the type of natural environments encountered, and the cultural roots of nature engagement on health outcomes remain poorly understood (46, 47). More research is needed to fully understand these complex relationships (55, 56). Further studies in regions with abundant natural spaces are necessary to generalize or contrast these findings more effectively. Longitudinal research could help determine whether participation in WAs predicts mental distress over time or if distressed adolescents are more likely to seek out nature. Investigating coping strategies in adolescents with mental distress who engage in WAs could provide valuable insights into the role of nature in their mental health and wellbeing. Additionally, distinguishing between structured vs. unstructured WAs and solitary vs. social participation could clarify the varying effects of exposure to nature on mental health. Comparative research with similar populations, particularly in other Nordic countries, would further contextualize these findings and help identify cultural and environmental factors influencing the WA-mental health relationship.

The detected associations with mental distress confirm that girls experience more problems than boys and suggest that mental health issues should be considered in relation to school achievement and adjustment. Adolescents who struggle to meet school demands appear to be at a higher risk of experiencing mental health issues that require attention, and vice versa. The complex relationships between mental health, wellbeing, and gender require further investigation.

## 5 Conclusion

This study sheds light on Norwegian adolescents' participation in WAs and its association with mental distress. The findings highlight the lower participation rates in WAs compared to national estimates, particularly lower levels among boys and children of foreign-born parents.

While WAs are widely promoted as beneficial, our findings suggest that their mental health effects are more nuanced than previously assumed. Authorities should be cautious about generalizing the benefits of WAs without considering individual differences in coping strategies, social engagement, and the emotional context of participation. Future research should continue to explore these complexities to ensure that WAs are leveraged as an effective tool for adolescent wellbeing. Finally, attention should be given to the growing influence of digital media on outdoor activity and its implications for adolescent wellbeing.

## Data Availability

The data analyzed in this study is subject to the following licenses/restrictions: the Trøndelag Health Study (HUNT) is a collaboration between HUNT Research Centre (Faculty of Medicine and Health Sciences, Norwegian University of Science and Technology NTNU), the Trøndelag County Council, Central Norway Regional Health Authority, and the Norwegian Institute of Public Health. Requests to access these datasets should be directed to Trøndelag Health Study (HUNT) (https://www.ntnu.edu/hunt/research).
